# Fatigue in acromegaly patients: a scoping review

**DOI:** 10.3389/fendo.2025.1601661

**Published:** 2025-07-14

**Authors:** Wei Wang, Xiaoxu Han, QingHua Huang

**Affiliations:** Department of Nursing, The Second Affiliated Hospital Zhejiang University School of Medicine, Hangzhou, China

**Keywords:** acromegaly, fatigue, scoping review, EBN, evidence-based nursing

## Abstract

**Purpose:**

This scoping review systematically examines current evidence on fatigue in acromegaly patients, with a particular focus on incidence, risk factors, hazards, assessment tools and therapeutic interventions, to inform evidence-based interventions aimed at improving rehabilitation outcomes.

**Methods:**

A scoping review was conducted following the PRISMA-ScR guidelines. We systematically searched five international databases (PubMed, Embase, CINAHL, Web of Science, and the Cochrane Library) and three Chinese databases (CNKI [China National Knowledge Infrastructure], WanFang, and Sinomed) from their inception through June 21, 2025. The inclusion criteria included original studies investigating fatigue manifestations in patients with acromegaly, including epidemiological studies, psychometric validation reports, and intervention trials. The exclusion criterion was articles focused exclusively on pharmacological or surgical interventions without fatigue assessment. Two independent researchers performed literature screening, data extraction, and quality appraisal via standardized protocols.

**Results:**

A total of 20 studies were included (all English-language publications). The prevalence of fatigue in acromegaly patients is high, ranging from 49% to 92%. Objective fatigue assessment tools primarily involve electromyography (EMG) and isokinetic dynamometry of the knee joint. Subjective fatigue was evaluated with patient self-reports. Factors that influence fatigue in these patients include demographic characteristics, sociological factors, sleep disturbances, comorbidities, and disease-specific factors. Interventions included aerobic exercise, rehabilitation therapist-guided home rehabilitation programs, and cognitive behavioral therapy.

**Conclusion:**

This scoping review underscores the need for future large-scale longitudinal studies on fatigue in acromegaly. Priority areas include identifying predictive markers, understanding pathophysiological mechanisms, evaluating targeted interventions, and developing standardized assessment tools to improve early recognition and management.

## Introduction

Acromegaly was first described by the French neurologist Pierre Marie in 1886 as a rare, chronic, and progressively debilitating endocrine disorder with an insidious onset (1). The disease is characterized by excessive secretion of growth hormone (GH) and elevated levels of insulin-like growth factor 1 (IGF-1), typically caused by a GH-secreting pituitary adenoma. The global prevalence of acromegaly is approximately 6 cases per 100,000 individuals, with an annual incidence of 2.8–4 cases per million population (2). Owing to advances in treatment modalities in recent years, the life expectancy of acromegaly patients has significantly increased (3).

Acromegaly is a specific form of pituitary adenoma, with early symptoms that are often nonspecific (e.g., joint pain, headaches, sensory disturbances, and excessive sweating) (4, 5), which may develop insidiously and progress slowly. As a result, there is often a delay of 5–10 years between the onset of symptoms and diagnosis (6). A qualitative interview study conducted in France revealed that, in some cases, the delay in diagnosis can be as long as 16 years (7). Prior to diagnosis, patients often undergo multiple consultations and diagnostic tests and suffer from a variety of comorbidities, which can lead to significant physical and mental fatigue. Fatigue is one of the most common symptoms among patients with acromegaly, affecting up to 53% of individuals and significantly impairing their quality of life (8).

Fatigue, while often conflated with general tiredness, is a distinct and complex symptom that significantly affects patients with chronic diseases, including acromegaly. Unlike transient tiredness, which typically resolves with rest, fatigue is persistent, often severe, and poorly relieved by sleep. Its characteristics span multiple domains: it tends to be long-lasting, intense and overwhelming and manifests both physically (e.g., muscle weakness, tremors) and mentally (e.g., lack of motivation, difficulty concentrating). The etiology of fatigue is multifactorial, potentially involving hormonal dysregulation, inflammation, comorbid depression, and disruptions in sleep patterns. Importantly, it exerts a profound impact on multiple levels of functioning—including occupational, social, and psychological domains—making it a central determinant of patients’ quality of life (4, 5).

Fatigue is a subjective symptom commonly characterized by persistent extreme tiredness or physical and mental fatigue, which is different from symptoms such as depression or muscle weakness. It is usually assessed through patient self-report questionnaires. Fatigue in patients is associated with increased morbidity, increased difficulty in disease management, decreased quality of life, and shortened life expectancy. With recent advancements in medical technology and the growing focus on rare diseases, comprehensive clinical and biochemical assessment criteria have been established. However, clinicians still pay insufficient attention to patients’ subjective experience, especially self-reported fatigue. Most existing assessment tools focus primarily on generic quality-of-life indicators. There is increasing evidence (9, 10)that biochemical control alone does not alleviate the clinical symptoms of these patients. Therefore, it is crucial to study fatigue in patients with acromegaly.

This study followed the methodological guidelines for scoping reviews outlined by Arksey and O’Malley (11) and aimed to collect and synthesize a wide range of information related to the core concept of ‘fatigue in patients with acromegaly’ without evaluating the methodological quality of individual studies. The primary objectives of this research are to explore the following issues: 1) the current state of fatigue in patients with acromegaly; 2) the factors contributing to fatigue and its associated adverse impacts; 3) the tools used for assessing fatigue; and 4) the interventions currently employed worldwide to manage fatigue in these patients.

## Methods

This scoping review is conducted following the framework of Arksey and O’Malley and the Preferred Reporting Items for Systematic Reviews and Meta-Analyses extension for Scoping Reviews (PRISMA-ScR) (11); the PRISMA-ScR checklist is presented in Appendix 1. This scoping review has not yet been registered.

The inclusion criteria were as follows: (1) Studies were included if participants with acromegaly accounted for ≥50% of the study sample; (2) studies focused on fatigue-related outcomes; (3) studies published in both Chinese or English; (4) original research articles.

The exclusion criteria were as follows: (1) studies for which full-text articles could not be obtained; (2) duplicate publications; and (3) newspaper articles, comments, and conference abstracts that were not included.

### Search strategy

We searched five international databases (PubMed, Embase, CINAHL, Web of Science, and the Cochrane Library) and three Chinese databases (CNKI, WanFang, and Sinomed) from inception to June 21, 2025. Furthermore, the reference lists of the included studies were systematically reviewed to identify any publications that may have been overlooked; these publications were then included in the analysis. No additional studies were identified in this research. The search process is shown in Appendix 2.

The retrieved literature was imported into Endnote X9 to remove duplicate studies. Two master’s students with evidence-based nursing training performed an initial screening based on the inclusion and exclusion criteria, followed by a full-text review for rescreening. If there was a disagreement, a third researcher was consulted to determine whether the study should be included. The researchers independently extracted relevant data from the included studies via a standardized form.

## Results

### Outcomes of the literature screening process

**Figure 1:**
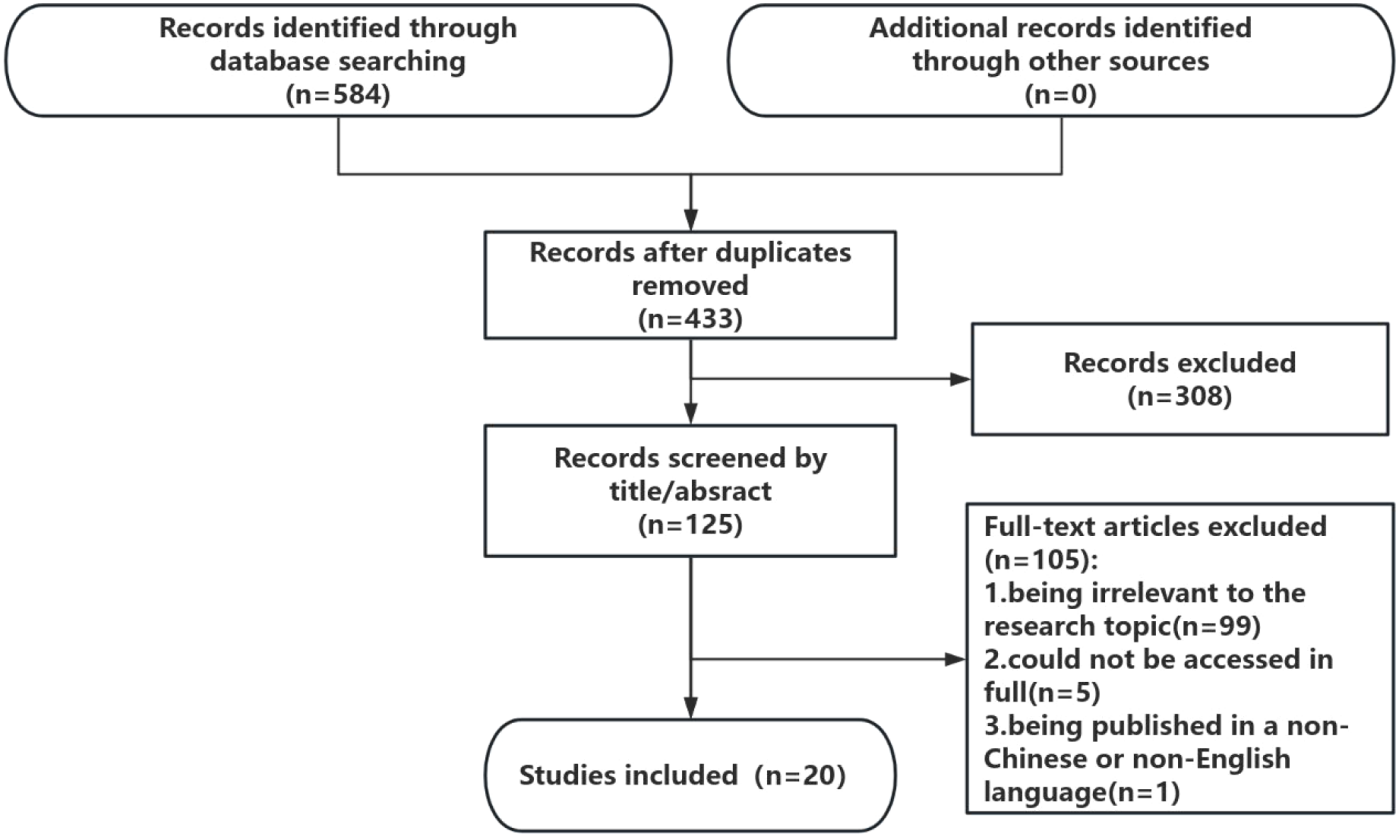
Flow chart of the study selection process.

A total of 584 articles were identified in the literature search. After deduplication, a total of 433 citations were identified from searches of electronic databases and references of review articles. On the basis of the title and abstract, 308 articles were excluded, and 125 full-text articles were retrieved and evaluated for eligibility. Of these, 105 were excluded for the following reasons: 99 studies were excluded for being irrelevant to the research topic, 5 articles could not be accessed in full, and one study was excluded because it was published in a non-Chinese or non-English language. The remaining 20 studies were considered to meet the requirements of this review. The literature selection process is shown in **Figure 1**.

### Basic characteristics of the included studies

A total of 20 studies focused on fatigue in patients with acromegaly, with the majority conducted in Brazil (n=7) and the Netherlands (n=3). Other contributing countries include the United States (n=2),China (n=1), France (n=2), Iraq (n=1), Turkey (n=1), Greece (n=1),Canada (n=1), India(n=1),representing 10 countries in total.

The basic characteristics of the included studies are provided in **Tables 1**, **2**.

**Table 1:** Basic characteristics of the included studies.

Author (year)	Country	Design	Sample (male/female)	Age (Years)	Therapy	Assessment tools	Distinctive Features of Fatigue
Agnaldo (2015) (12)	Brazil	Cross-sectional	32 (exact M/F not specified)	48.6 ± 12.1	–	6MWD	Fatigue in peripheral muscles, fat-free mass (FFM), and maximal expiratory pressure (MEP) are key determinants of the 6-minute walk distance (6MWD) in patients with acromegaly.
Biermasz等 (2005) (13)	Netherlands	Cross-sectional	118 (61/57)	58.61 ± 12.9	surgery, radiotherapy, and/or somatostatin analog treatment	MFI-20	Patients with a history of myocardial infarction tend to score lower in general health, depression, and fatigue. In individuals with acromegaly, joint-related complications significantly impact the level of fatigue, leading to a reduction in their quality of life.
Biermase (2004) (14)	Netherlands	Cross-sectional	118 (61/57)	58.6 ± 12.9	surgery, radiotherapy, and/or somatostatin analog treatment	MFI	Radiation therapy-induced physical fatigue serves as an independent predictor of reduced quality of life in patients.
Martin (2023) (15)	American	Qualitative interview	16 (5/11)	47.3 (24,63)	_	ASD	Develop the Acromegaly Symptom Diary (ASD), establish a scoring algorithm, and evaluate the psychometric measurement properties of the ASD.
Lin (2023) (4)	China	Cross-sectional	106 (52/54)	–	Surgery (n=30), medication and surgery (n=76)	PASQ	Decreased hormone levels in patients are a significant contributing factor to fatigue.
Homem (2017) (16)	Brazil	Case–Control Study	Acromegaly patients (n=26);healthy elderly Subjects (n=20)	67 (63,73); 64 (61,68)	_	–	Compared to healthy elderly individuals, older adults with acromegaly are more prone to fatigue.
Agatha A (2008) (17)	Netherlands	Cross-sectional	The group of Acromegaly :118 (57/61)	58.6 ± 12.9	Surgery (n= 108) Radiotherapyn=33)	MFI-20	The prevalence of fatigue is high in acromegaly patients during long-term follow-up after surgery.
Michalski (2019) (18)	Brazil	Cross-sectional	23 (14/9)	49.4 ± 11.2	_	MFIS、6MWT	Hormonal, vascular, and functional variables can predict general fatigue in patients with acromegaly.
Alzajaji (2021) (9)	Iraq	Cross-sectional	56 (24/32)	47.8 ± 11.3	_	_	The incidence of fatigue in acromegaly patients is as high as 82%, and its severity is unrelated to biochemical control.
Albarel (2019) (19)	France	Cross-sectional	28 (exact M/F not specified)	48.9	drug therapy	chief complaint	According to the survey, 54% of patients reported experiencing fatigue as a primary complaint.
Ozturk (2020) (20)	Turkey	Prospective case–control study	Acromegaly patients (n=39); healthy control subjects (n=39)	_	_	MAF、GFI	Acromegaly results in increased thickness of tendons and soft tissues, reduced muscle thickness, and deterioration of lower limb microstructure, all of which contribute to muscle fatigue in affected patients.
Guedes (2013) (21)	Brazil	Cross-sectional	32 subjects with acromegaly (10/22)	50 (44.5-55.8)	Surgery (n=21); radiotherapy (n=8)*	EMG	The study employed electromyography (EMG) to assess fatigue in patients with acromegaly.”
Linda J Woodhouse (2006) (22)	Canada	Report	_	_	_	_	Fatigue in patients with acromegaly is associated with reduced physical activity levels.
Anagnostis (2014) (23)	Greece	Cross-sectional	40	60 ± 1.96	_	the Greek-brief version of POMS ; AcroQoL	In terms of gender differences in acromegaly patients, female patients are more likely to experience fatigue.
Shah (2025) (24)	United States	Qualitative, community meeting summary	~50 participants (exact M/F not specified)	_	Surgery (83%); medical treatment (71%)	No formal tools; data derived from structured patient feedback during community meeting	Fatigue described as the most common and distressing symptom (92%); includes muscle weakness, impacts on daily living, and persisting even with treatment; fatigue affected physical activity, work, social life, and psychological well-being
Caron (2019) (25)	France	Observational, cross-sectional, multicenter non-interventional	N = 472 (sex breakdown not specified)	Adults (diagnosed <5 years); precise age not reported	Variable: some had prior pituitary surgery and/or medical treatment	Patient questionnaires and clinical case-report forms (CRF)	Asthenia reported by 79.2 % of patients at diagnosis.
Hashim (2022) (26)	India	Prospective, observational	N = 35	39.7 ± 13.2	Active acromegaly (likely untreated/untreated)	Respiratory symptoms questionnaire (self-reported)Epworth Sleepiness Scale (ESS)	49% of patients reported fatigue as a frequent symptom; fatigue often co-occurred with daytime somnolence, dyspnea, and cough, indicating a significant disease burden

6MWT, Six-minute walk test; MFI-20, Multidimensional Fatigue Index 20; ASD, Acromegaly Symptom Diary; PASQ, Patient-Assessed Acromegaly Symptom Questionnaire; MFIS, Modified Fatigue Impact Scale; EMG, Electromyography; MAF, Multidimensional Assessment of Fatigue; GFI, Global Fatigue Index; EMG, POMS, Profile of Mood States; AcroQol, Acromegaly Quality of Life; CRF, Case Report Form.

*Some patients received combined treatments, while others with controlled disease did not receive any treatment.

**Table 2:** Characteristics of the included intervention studies.

Author	Country	Design	Sample	Type of intervention	Content of intervention	Dose	Providers	Control	Duration	Outcomes
Lima(2019) (3)	Brazil	Self comparison before and after	17	Therapist oriented home rehabilitation	5 min of warm-up exercises, 20 min of Muscle strengthening and resistance exercises for the upper and lower limbs, 10 min of balance training, 20 min of aerobic training, 5 min of global stretching and relaxation exercises.	Before starting the protocol, the patients were instructed by how to perform the physical exercises, and followed an Exercise programme from a booklet with instructions for each exercise prescribed for 3 times a week, for a total of 24 sessions.	Physiotherapist	_	2months	Quality of life, fatigue, body com position, handgrip strength, lower extremity functional ity, body balance and functional capacity
Lia Silvia Kunzler (2018) (27)	Brazil	Nonran domized clinical trial	20	Cognitivebehavioral therapy (CBT)	Basic concepts of CBT; emotional regulation;increase self-esteem and self-confidence, and supply of a brain-shaped piggy bank; “Think healthy and feel the difference”technique; the technique for approaching anger, assertiveness training, and the coping card; the technique for approaching fear; the technique adapted for shame about one’s Physical appearance in acromegaly; clarifications on acromegaly; a summary of the topics covered in the sessions.	Nine weekly 90-min” Think Healthy” group therapy sessions.	_	_	3months	The quality of life in patients with acromegaly has improved.
Lemos (2019) (28)	Brazil	case study	1	Therapist oriented home rehabilitation	Activities included overall stretching and strengthening (flexion, extension, adduction and abduction movements) and muscular endurance exercises (exercises involving open and closed kinetic chains), along with aerobic conditioning using a functional circuit.	the patient underwent a booklet-guided physical exercise program that lasted two months (3 times per week with a duration of 60 minutes per session).	Physiotherapist	_	2months	HRQoL, general fatigue, peripheral muscle strength and functional capacity assessments

### Prevalence of fatigue in patients with acromegaly

The prevalence of fatigue in patients with acromegaly is high, ranging from 49% to 92% (19, 25, 26) Studies that met the inclusion criteria reported 5 types of fatigue factors.

### General demographic factors

In terms of sex differences in acromegaly patients, female patients are more likely to experience fatigue (23).

### Sociopsychological factors

Some scholars believe that the chronic fatigue experienced by patients with acromegaly may be associated with a modern sedentary lifestyle, which is characterized by low physical activity and self-circulation. Additionally, reduced physical exertion after surgery may exacerbate subjective fatigue-a feedback loop may develop: fatigue leads to reduced physical activity, which in turn exacerbates deconditioning and further augments fatigue (22). Patients often experience decreased activity and periods of immobility after pituitary or other major surgeries. Such inactivity can lead to rapid muscle atrophy and loss of endurance—processes collectively referred to as physical deconditioning—which are well-documented contributors to increased fatigue levels (29). On the other hand, delayed diagnosis and treatment of the disease often result in decreased social participation, heightened psychological uncertainty, and a heavy mental burden, all of which may contribute to fatigue in patients (30).

### Sleep disorders/metabolic abnormalities

Obstructive sleep apnea syndrome is frequently observed in patients with active acromegaly, with a prevalence of approximately 69% (26). Although reductions in GH/IGF-1 levels can alleviate the severity of sleep apnea, up to 40% of acromegaly patients still experience persistent sleep apnea. Sleep deprivation caused by apnea may lead to the onset of chronic fatigue syndrome (22). Additionally, the enormous psychological burden on patients may cause them to fall asleep, while visual impairment may further affect sleep quality and aggravate fatigue (31).

### Comorbidities

One study (13) indicated that patients with a history of myocardial infarction have a greater risk of developing fatigue. Acromegaly patients also experience a high and irreversible incidence of joint-related complications, which can significantly affect the severity of fatigue and reduce their quality of life.

### Disease-specific factors

1) Fatigue in patients with acromegaly is significantly associated with elevated expression of proinflammatory cytokines in the cortex and hippocampus (28). 2) Abnormal hormone secretion: Studies have demonstrated that hormonal levels serve as independent predictors of general fatigue in patients with acromegaly (4, 18). Preoperative fasting GH levels are significant determinants of fatigue severity in these individuals, whereas improvements in biochemical markers are not positively correlated with self-reported fatigue (9). 3) Vascular damage: The increased risk of peripheral vascular disease progressively impairs the adaptive perfusion of skeletal muscle microcirculation. Vascular damage contributes to alterations in muscle fiber composition and dysfunction, which in turn leads to increased fatigue scores in acromegaly patients (18). 4) Acromegaly causes thickening of tendons and soft tissues, decreased muscle thickness, and deterioration of the microstructure of the lower limb, all of which lead to muscle fatigue in patients (20). 5) Treatment-related factors: Radiation therapy-induced physical fatigue has been identified as an independent predictor of decreased quality of life in patients (14).

### The impact of fatigue on health outcomes in patients with acromegaly

Two studies (12, 14) reported the impact of fatigue on health outcomes in patients with acromegaly. 1) Decreased quality of life: One study showed that fatigue caused by radiotherapy was an independent predictor of decreased quality of life in patients (14). 2) Skeletal muscle fatigue: Fatigue in skeletal muscles can limit exercise tolerance in various clinical conditions, with quadriceps fatigue identified as one of the primary determinants of poor performance in the 6-minute walk test (12).

### Assessment tools for fatigue in acromegaly patients

The studies included in this study primarily employed objective fatigue assessment tools related to electromyography (EMG) and knee isokinetic measurements, alongside patient-reported subjective fatigue assessment scales.

### Objective assessment tools

Two studies (12, 21) employed surface electromyography (sEMG) to assess muscle fatigue in patients with acromegaly. The electromyographic recordings provide two key parameters: the root mean square (RMS), which quantifies electrical activity during muscle contraction, and the median frequency (MDF), which reflects the frequency of motor unit action potentials. Muscle fatigue is positively correlated with the RMS slope and negatively correlated with the MDF slope. Therefore, higher RMS values indicate more severe muscle fatigue, and higher MDF values ​​indicate less fatigue.

This study (16) utilized knee isokinetic measurements and a dynamometer to assess major lower extremity muscle groups. The patient sat on the dynamometer with the trunk, pelvis, and thighs secured with straps. Prior to the formal assessment, the torque produced by the weight of the lower limb at a 5° knee flexion position was measured to correct for the effects of gravity on the affected muscles. The patient then underwent three practice trials to familiarize themselves with the dynamometer. Strength and endurance analyses were conducted separately, allowing for the calculation of the patient’s fatigue index.

### Subjective assessment tools

Studies (14, 17) have employed the Multidimensional Fatigue Inventory-20 (MFI-20) to assess fatigue in patients. This scale was developed by a research group in the Netherlands in 1995 (32) and includes five dimensions, namely, general fatigue, physical fatigue, reduced activity, reduced motivation, and mental fatigue, with a total of 20 items. Fatigue is assessed via a 5-point Likert scale (14), with scores ranging from 0–20, where higher scores indicate greater fatigue severity. The Chinese version of the MFI-20 has been validated in Chinese cancer patient populations, with a reliability coefficient of 0.89, making it a reliable and effective tool for fatigue assessment (33). Due to the rarity of the disease, the application of this scale in acromegaly patients is still limited.

Study (18) used the Modified Fatigue Impact Scale (MFIS) for assessment. The total score range is 0–84, encompassing three dimensions—cognitive, physical, and psychosocial functioning—with a total of 21 items. The lower the score is, the milder the degree of fatigue, and the cutoff value for distinguishing between fatigued and nonfatigued individuals is 38 points.

Studies (3, 28) have employed the Functional Assessment of Chronic Illness Therapy-Fatigue (FACIT-F) scale, which consists of 13 items, with scores ranging from 0–4. A higher score indicates a greater degree of fatigue. The FACIT-F is a specific scale designed for fatigue assessment and encompasses four dimensions: physical fatigue (e.g., ‘I feel tired’), functional fatigue (e.g., ‘I find it hard to complete tasks’), emotional fatigue (e.g., ‘I feel depressed’), and the social consequences of fatigue (e.g., ‘It limits my social activities’).

One study (4) used the Patient-Assessed Acromegaly Symptom Questionnaire (PASQ) to measure fatigue in patients with acromegaly. This questionnaire was first proposed by Trainer et al. in 2000 and is a disease-specific tool designed to assess the severity of patient-reported symptoms, including five key symptoms (soft tissue swelling, joint pain, headaches, excessive sweating, and fatigue). The scoring ranges from 0 to 8, with 0 indicating no symptoms and 8 indicating severe disability due to symptoms. The total PASQ score is the sum of the scores for each individual symptom, with a maximum possible score of 40. In general, the higher the PASQ score is, the more difficult it is to tolerate symptoms. This scale is the most widely used disease-specific assessment tool for acromegaly patients.

A study (15) conducted semistructured interviews with 16 adult patients with acromegaly, combining concept-driven and cognitive reporting methods to develop the Acromegaly Symptom Diary (ASD). This diary includes 9 symptoms related to acromegaly: headaches, joint pain, sweating, fatigue, leg weakness, swelling, numbness/tingling, sleep disturbances, and short-term memory difficulties. The ASD primarily assesses symptoms experienced by acromegaly patients in the past 24 hours on an 11-point scale ranging from 0 (no symptoms) to 10 (worst symptoms). The ASD allows for an effective and reliable measurement of the severity of acromegaly symptoms. The key distinction between this tool and other patient-reported fatigue scales is the 24-hour recall period for reporting symptoms, which accounts for symptom fluctuations, especially those affected by medications.

### Intervention methods for fatigue in acromegaly patients

Aerobic exercise: One study (3) conducted a prospective intervention with 17 acromegaly patients who followed an exercise regimen guided by a physical therapist. The programme consisted of strength, aerobic, and flexibility exercises performed three times a week for 60 minutes for two months. The outcome measures included general fatigue, quality of life, and various physical assessments, such as grip strength, lower limb muscle strength, static body balance, and walking tests. The study revealed that aerobic exercise improved quality of life and reduced fatigue in acromegaly patients. It was suggested that aerobic exercise could serve as an adjunctive therapy for the biochemical control of acromegaly, contributing to overall improvements in quality of life.

Cognitive behavioral therapy (CBT): One study (27) reported that CBT could improve fatigue symptoms in acromegaly patients. While previous research [32] has confirmed the effectiveness of CBT in improving chronic fatigue syndrome in patients with other systemic diseases, research in acromegaly patients is limited. A Brazilian research team was the first to apply CBT to the acromegaly patient population. The intervention took place in a quiet and private room, with participants receiving ‘Think Healthy’ group therapy, which was delivered weekly for 9 sessions, each lasting 90 minutes. ‘Think Healthy’ is a mobile application available for download on both the Apple Store and Google Play, with versions in both Portuguese and English. The study revealed that the ‘Think Healthy’ CBT technique improved the quality of life of acromegaly patients. However, the study used the SF-36 (Quality of Life Questionnaire) to assess overall quality of life as the primary outcome, which, while related to quality of life, has limited specificity for fatigue assessment. Future studies could utilize fatigue-specific assessment scales for further validation.

Therapist-oriented home rehabilitation (TOHR): One study (28) confirmed the effectiveness of therapist-oriented home rehabilitation (TOHR) for acromegaly patients. The study noted that after surgery combined with medical treatment, patients were able to live independently but still reported persistent symptoms such as fatigue and pain. Consequently, after a physical therapy assessment, patients were enrolled in a two-month physical exercise program that included overall stretching and strengthening (flexion, extension, adduction, and abduction exercises), muscle endurance training (including both open and closed kinetic chain exercises), and aerobic conditioning via functional cycling. The study revealed that acromegaly patients could benefit significantly from TOHR, which can be used as an adjunct to hormone-controlled therapy.

### Discussion

#### Multidimensional nature and clinical neglect of fatigue in acromegaly

Although acromegaly is a low-prevalence disease, it can lead to multisystem involvement once it occurs and is accompanied by multiple complications, a reduced quality of life, and an increased incidence of fatigue. Fatigue in acromegaly patients is multidimensional, encompassing both physical and psychological fatigue (22). However, the underlying physiological and pathological mechanisms that contribute to fatigue in acromegaly patients remain unclear (34). A qualitative interview study conducted in the United States with patients suffering from rare diseases revealed that the most frequently reported symptoms were fatigue and pain. The prolonged inability to diagnose and treat the disease has led to reduced social participation, psychological uncertainty, and a heavy mental burden, all of which contribute to the experience of fatigue. However, very few patients receive specialized interventions (30). Currently, the treatment strategy for acromegaly primarily involves surgery and pharmacological therapy aimed at improving biochemical markers. Fatigue is among the most prominent complaints reported by patients, but it is often ignored by healthcare providers. Few studies focusing on subjective fatigue have examined it as a single dimension without distinguishing between physical and psychological fatigue (35).

Fatigue is the most frequently reported and severe physical symptom by patients, who describe it as ‘extreme chronic fatigue,’ often accompanied by ‘physical trembling,’ significantly impacting their quality of life (35). In studies, fatigue is monitored mainly through patient self-reports, with a high incidence (4). A qualitative interview study in the United States focusing on rare disease patients revealed that the symptoms most commonly reported by patients were fatigue and pain. Owing to prolonged diagnostic delays and a lack of treatment, patients experience reduced social participation, heightened psychological uncertainty, and heavy mental burdens, leading to fatigue. However, very few patients receive specialized interventions (28).

In addition to physiological mechanisms, psychological fatigue should be acknowledged as a relevant and impactful component among patients with acromegaly. Psychological fatigue is characterized by diminished resilience to stress, emotional exhaustion, reduced motivation, and decreased subjective well-being. Patients may experience altered personality traits, such as increased irritability or withdrawal, and struggle with adapting to the long-term burden of a chronic endocrine disorder (36). These manifestations, though less visible than physical symptoms, are no less disabling.

Furthermore, the cultural context appears to play a role in how fatigue is perceived, expressed, and managed. A comparison of Chinese and Western cohorts in this review highlights both shared patterns and notable differences. For instance, Chinese patients may report fatigue less frequently or describe it in somatic terms due to cultural norms surrounding emotional expression, whereas Western cohorts may articulate psychological aspects more explicitly. Healthcare access, social support systems, and stigma associated with chronic fatigue or depression may also differ between regions, influencing both prevalence data and the interpretation of patient-reported outcomes (37).

Future studies should explore these cultural and psychosocial dimensions in greater depth to develop more culturally sensitive assessment tools and interventions.

#### Fatigue assessment tools

Fatigue in studies has been monitored mainly through patient self-reports, with a high incidence observed (8, 9, 19). Fatigue assessment in patients with acromegaly includes both objective tools, such as electromyography (EMG) and knee joint isokinetic measurements, as well as subjective self-reported tools. Owing to potential limitations in the objective conditions of studies, the majority of the current literature relies on patient self-reported assessments. Among these, the MFI-20 and FACIT-F are the most commonly used, and their effectiveness in evaluating fatigue may warrant further validation in future studies. The PASQ is a tool specifically designed for symptom assessment in patients with acromegaly that is currently underutilized.

In addition to the general mechanisms contributing to fatigue in acromegaly, disease-specific factors such as anemia and hypogonadism have been increasingly recognized as important contributors. Anemia may exacerbate fatigue by reducing oxygen delivery to tissues, thereby impairing cellular energy metabolism and leading to increased physical exhaustion. Hypogonadism, commonly observed in patients with acromegaly due to pituitary dysfunction, can further aggravate fatigue through hormonal imbalances that affect muscle strength, mood, and overall vitality. These conditions not only reflect the systemic impact of the disease but also may persist despite biochemical control of growth hormone and IGF-1 levels, thus representing potential targets for adjunctive management strategies. Incorporating assessment and treatment of anemia and hypogonadism into the comprehensive care of acromegaly patients may help alleviate fatigue and improve quality of life (38, 39).

Although aerobic exercise programs have been shown to alleviate fatigue in patients, such programs must be tailored to the individual, taking into account hormone levels and the degree of biochemical control. Comprehensive assessment and treatment of clinical symptoms should be an important part of the management plan for acromegaly patients.

Currently, the main treatment strategies for acromegaly focus on surgery and pharmacotherapy to improve biochemical markers, but fatigue, the most prominent complaint of patients, is often overlooked by healthcare providers. A few studies limit subjective fatigue to a single dimension and are unable to clearly distinguish between physical fatigue and psychological fatigue. Although aerobic exercise programs have been shown to improve fatigue in patients, these programs must be tailored to individual patients, taking into account hormone levels and the degree of biochemical control achieved. Comprehensive evaluation and treatment of clinical symptoms are important parts of the management plan for acromegaly patients.

### Limitations

Although this study conducted a rigorous process of literature search and data selection, existing studies on fatigue assessment in patients with acromegaly often combine fatigue with quality of life assessment. Many studies have indirectly confirmed the occurrence of fatigue through decreased quality of life, and such studies were not included in this study. Furthermore, fatigue in patients with acromegaly may include both physical and psychological components. Although this study involved these two aspects, there may still be gaps in the covered literature. Finally, our literature search was limited to studies published in English and Chinese, which may introduce publication bias.

## Conclusions

Healthcare providers should recognize the impact of fatigue on the physical recovery and quality of life of acromegaly patients. In addition, risk factors associated with fatigue, including disease-specific factors, sleep disorders, and comorbidities, should be identified. Therefore, it is recommended that fatigue be incorporated into the routine perioperative assessment of acromegaly patients. Fatigue should be evaluated both preoperatively and postoperatively, with a focus on tracking changes in fatigue levels. These findings will provide valuable data for future studies on fatigue trajectories in these patients and facilitate timely identification and intervention, ultimately promoting faster recovery.

## References

[1] Castellanos-Bueno R Abreu-Lomba A Buitrago-Gómez N Patiño-Arboleda M Pantoja-Guerrero D Valenzuela-Rincón A . Clinical and epidemiological characteristics, morbidity and treatment based on the registry of acromegalic patients in Colombia: RAPACO. Growth Hormone IGF Res. (2021) 60-61:101425. doi: 10.1016/j.ghir.2021.101425, PMID: 34416544

[2] Brue T Rahabi H Barry A Barlier A Bertherat J Borson-Chazot F . Position statement on the diagnosis and management of acromegaly: The French National Diagnosis and Treatment Protocol (NDTP). Ann Endocrinol (Paris). (2023) 84:697–710. doi: 10.1016/j.ando.2023.08.003, PMID: 37579837

[3] Lima T Kasuki L Gadelha M Lopes AJ . Physical exercise improves functional capacity and quality of life in patients with acromegaly: a 12-week follow-up study. Endocrine. (2019) 66:301–9. doi: 10.1007/s12020-019-02011-x, PMID: 31317523

[4] Lin B He W Chen Z Shen M Shou X Chen L . Self-reported symptoms in patients with acromegaly: a 6-month follow-up in a single neurosurgical center. Endocr. J. (2023) 70:77–87. doi: 10.1507/endocrj.EJ22-0241, PMID: 36198614

[5] Unger N Theodoropoulou M Schilbach K . Clinically active pituitary tumors. INNERE Med. (2024) 65:672–80. doi: 10.1007/s00108-024-01729-9, PMID: 38869654

[6] Crisafulli S Fontana A L’Abbate L Vitturi G Cozzolino A Gianfrilli D . Machine learning-based algorithms applied to drug prescriptions and other healthcare services in the Sicilian claims database to identify acromegaly as a model for the earlier diagnosis of rare diseases. Sci Rep. (2024) 14:6186. doi: 10.1038/s41598-024-56240-w, PMID: 38485706 PMC10940660

[7] Sibeoni J Manolios E Verneuil L Chanson P Revah-Levy A . Patients’ perspectives on acromegaly diagnostic delay: a qualitative study. Eur J Endocrinol. (2019) 180:339–52. doi: 10.1530/EJE-18-0925, PMID: 30939451

[8] Slagboom T van Bunderen CC De Vries R Bisschop PH Drent ML . Prevalence of clinical signs, symptoms and comorbidities at diagnosis of acromegaly: a systematic review in accordance with PRISMA guidelines. Pituitary. (2023) 26:319–32. doi: 10.1007/s11102-023-01322-7, PMID: 37210433 PMC10397145

[9] Alzajaji QB Alidrisi HA Mansour AA . Correlation between clinical and biochemical markers in patients with acromegaly on different modalities of treatment. Cureus. (2021) 13:e19438. doi: 10.7759/cureus.19438, PMID: 34909342 PMC8663996

[10] Geer EB Sisco J Adelman DT Ludlam WH Haviv A Liu S . Patient reported outcome data from acromegaly patients treated with injectable somatostatin receptor ligands (SRLs) in routine clinical practice. BMC Endocr Disord. (2020) 20:117. doi: 10.1186/s12902-020-00595-4, PMID: 32736547 PMC7393879

[11] Arksey H O’Malley L . Scoping studies: towards a methodological framework. Int J Soc Res Method. (2005) 8:19–32. doi: 10.1080/1364557032000119616

[12] Lopes AJ Guedes Da Silva DP Ferreira ADS Kasuki L Gadelha MR Guimaraes FS . What is the effect of peripheral muscle fatigue, pulmonary function, and body composition on functional exercise capacity in acromegalic patients? J Phys Ther Sci. (2015) 27:719–24. doi: 10.1589/jpts.27.719, PMID: 25931716 PMC4395700

[13] Biermasz NR Pereira AM Smit JW Romijn JA Roelfsema F . Morbidity after long-term remission for acromegaly: persisting joint-related complaints cause reduced quality of life. J Clin Endocrinol Metab. (2005) 90:2731–9. doi: 10.1210/jc.2004-2297, PMID: 15741257

[14] Biermasz NR van Thiel SW Pereira AM Hoftijzer HC van Hemert AM Smit JW . Decreased quality of life in patients with acromegaly despite long-term cure of growth hormone excess. J Clin Endocrinol Metab. (2004) 89:5369–76. doi: 10.1210/jc.2004-0669, PMID: 15531483

[15] Martin S Bender RH Krasner A Marmon T Monahan M Nelson L . Development and evaluation of the acromegaly symptom diary. J patient-reported outcomes. (2023) 7:15. doi: 10.1186/s41687-023-00541-7, PMID: 36792844 PMC9931976

[16] Homem TS Guimaraes FS Soares MS Kasuki L Gadelha MR Lopes AJ . Balance control and peripheral muscle function in aging: A comparison between individuals with acromegaly and healthy subjects. J Aging Phys Activ. (2017) 25:218–27. doi: 10.1123/japa.2016-0100, PMID: 27622780

[17] van der Klaauw AA Kars M Biermasz NR Roelfsema F Dekkers OM Corssmit EP . Disease-specific impairments in quality of life during long-term follow-up of patients with different pituitary adenomas. Clin Endocrinol. (2008) 69:775–84. doi: 10.1111/j.1365-2265.2008.03288.x, PMID: 18462264

[18] Michalski ADC Ferreira ADS Kasuki L Gadelha MR Lopes AJ Guimaraes FS . Clinical and functional variables can predict general fatigue in patients with acromegaly: an explanatory model approach. Arch Endocrinol Metab. (2019) 63:235–40. doi: 10.20945/2359-3997000000127, PMID: 31038594 PMC10522193

[19] Albarel F Elaraki F Delemer B . Daily life, needs and expectations of patients with acromegaly in France: An on-line survey. Ann Endocrinol-Paris. (2019) 80:110–6. doi: 10.1016/j.ando.2018.08.006, PMID: 30612694

[20] Ozturk Gokce B Gogus F Bolayir B Tecer D Gokce O Eroglu Altinova A . The evaluation of the tendon and muscle changes of lower extremity in patients with acromegaly. Pituitary. (2020) 23:338–46. doi: 10.1007/s11102-020-01037-z, PMID: 32200458

[21] Guedes DSD Guimarães FS Dias CM Guimarães SA Kasuki L Gadelha MR . On the functional capacity and quality of life of patients with acromegaly: are they candidates for rehabilitation programs? J Phys Ther Sci. (2013) 25:1497–501. doi: 10.1589/jpts.25.1497, PMID: 24396219 PMC3881486

[22] Woodhouse LJ Mukherjee A Shalet SM Ezzat S . The influence of growth hormone status on physical impairments, functional limitations, and health-related quality of life in adults. Endocr Rev. (2006) 27:287–317. doi: 10.1210/er.2004-0022, PMID: 16543384

[23] Anagnostis P Efstathiadou ZA Charizopoulou M Selalmatzidou D Karathanasi E Poulasouchidou M . Psychological profile and quality of life in patients with acromegaly in Greece. Is there any difference with other chronic diseases? Endocrine. (2014) 47:564–71. doi: 10.1007/s12020-014-0166-5, PMID: 24510628

[24] Shah SN Yuen K Bonert V Huang W Sisco J Palaty C . Patient perspectives on acromegaly disease burden: insights from a community meeting. Front Endocrinol (Lausanne). (2025) 16:1516131. doi: 10.3389/fendo.2025.1516131, PMID: 39963277 PMC11830622

[25] Caron P Brue T Raverot G Tabarin A Cailleux A Delemer B . Signs and symptoms of acromegaly at diagnosis: the physician’s and the patient’s perspectives in the ACRO-POLIS study. Endocrine. (2019) 63:120–9. doi: 10.1007/s12020-018-1764-4, PMID: 30269264 PMC6329724

[26] Hashim Z Gupta M Nath A Khan A Neyaz Z Tiwari S . Prevalence of sleep apnea and lung function abnormalities in patients with acromegaly. Lung India. (2022) 39:58–64. doi: 10.4103/lungIndia.lungIndia_182_21, PMID: 34975054 PMC8926218

[27] Kunzler LS Naves LA Casulari LA . Cognitive-behavioral therapy improves the quality of life of patients with acromegaly. Pituitary. (2018) 21:323–33. doi: 10.1007/s11102-018-0887-1, PMID: 29644512

[28] Lemos Lima TR Kasuki L Gadelha MR Lopes AJ . The effectiveness of a therapist-oriented home rehabilitation program for a patient with acromegaly: A case study. J Bodywork Movement Therapies. (2019) 23:634–42. doi: 10.1016/j.jbmt.2019.01.006, PMID: 31563382

[29] Bazelmans E Bleijenberg G van der Meer JW Folgering H . Is physical deconditioning a perpetuating factor in chronic fatigue syndrome? A controlled study on maximal exercise performance and relations with fatigue, impairment and physical activity. Psychol Med. (2001) 31:107–14. doi: 10.1017/s0033291799003189, PMID: 11200949

[30] Mease C Fermaglich LJ Jackler K Shermer S Miller KL . Determining commonalities in the experiences of patients with rare diseases: A qualitative analysis of US food and drug administration patient engagement sessions. Patient. (2024) 17:25–37. doi: 10.1007/s40271-023-00648-5, PMID: 37833521 PMC11841686

[31] Andela CD Niemeijer ND Scharloo M Tiemensma J Kanagasabapathy S Pereira AM . Towards a better quality of life (QoL) for patients with pituitary diseases: results from a focus group study exploring QoL. Pituitary. (2015) 18:86–100. doi: 10.1007/s11102-014-0561-1, PMID: 24682940

[32] Smets EM Garssen B Bonke B De Haes JC . The Multidimensional Fatigue Inventory (MFI) psychometric qualities of an instrument to assess fatigue. J Psychosom Res. (1995) 39:315–25. doi: 10.1016/0022-3999(94)00125-o, PMID: 7636775

[33] Tian J Hong JS . Validation of the Chinese version of Multidimensional Fatigue Inventory-20 in Chinese patients with cancer. Support. Care Cancer. (2012) 20:2379–83. doi: 10.1007/s00520-011-1357-8, PMID: 22198167

[34] Hong CS Smith TR . Aerobic exercise interventions to address impaired quality of life in patients with pituitary tumors. PLoS One. (2023) 18(12):e0295907. doi: 10.1371/journal.pone.0295907, PMID: 38100429 PMC10723697

[35] Andela CD Scharloo M Ramondt S Tiemensma J Husson O Llahana S . The development and validation of the Leiden Bother and Needs Questionnaire for patients with pituitary disease: the LBNQ-Pituitary. Pituitary. (2016) 19:293–302. doi: 10.1007/s11102-016-0707-4, PMID: 26809957 PMC4858557

[36] Voss S Boachie DA Nieves N Gothe NP . Mind-body practices, interoception and pain: a scoping review of behavioral and neural correlates. Ann Med. (2023) 55:2275661. doi: 10.1080/07853890.2023.2275661, PMID: 37939212 PMC10768869

[37] Cui S Chen S Wu X Wang Q . Research status and prospects of pituitary adenomas in conjunction with neurological and psychiatric disorders and the tumor microenvironment. Front Neurosci. (2024) 18:1294417. doi: 10.3389/fnins.2024.1294417, PMID: 38716256 PMC11075501

[38] Palacios JD Komotar RJ Kargi AY . Successful treatment of acromegaly and associated hypogonadism with first-line clomiphene therapy. Case Rep Endocrinol. (2018) 2018:7925019. doi: 10.1155/2018/7925019, PMID: 30046497 PMC6038655

[39] Paixão Côrtes Aguiar D Chitolina Nesello A Araujo B Mafalda Dos Santos T Diniz Lima V Semmelmann Pereira Lima JF . Anemia in patients with pituitary adenomas: prevalence and correlation with hypopituitarism. Cureus. (2025) 17(6):e85422. doi: 10.7759/cureus.85422, PMID: 40621322 PMC12228548

