# Supplementary material for: Fatigue in acromegaly patients: a scoping review
Source: Front Endocrinol (Lausanne). 2025 Jul 14;16:1601661. doi: 10.3389/fendo.2025.1601661 (PMC12301152; doi:10.3389/fendo.2025.1601661)
Supplement: Supplementary file 1 [file DataSheet1.docx]

Appendix 1:PRISMA-ScR checklist

| Section | Item | PRISMA-ScR Checklist Item |
| --- | --- | --- |
| Title | 1 | **Fatigue in acromegaly patients: a scoping review** |
| Abstract | 2 | Purpose: This scoping review systematically examines current evidence on fatigue in acromegaly patients, with a particular focus on incidence, risk factors, hazards, assessment tools and therapeutic interventions, to inform evidence-based interventions aimed at improving rehabilitation outcomes.  Methods: A scoping review was conducted following the PRISMA-ScR guidelines. We systematically searched five international databases (PubMed, Embase, CINAHL, Web of Science, and the Cochrane Library) and three Chinese databases (CNKI [China National Knowledge Infrastructure], WanFang, and Sinomed) from their inception through June 21, 2025. The inclusion criteria included original studies investigating fatigue manifestations in patients with acromegaly, including epidemiological studies, psychometric validation reports, and intervention trials. The exclusion criterion was articles focused exclusively on pharmacological or surgical interventions without fatigue assessment. Two independent researchers performed literature screening, data extraction, and quality appraisal via standardized protocols.  Results: A total of 20 studies were included (all English-language publications). The prevalence of fatigue in acromegaly patients is high, ranging from 49% to 92%. Objective fatigue assessment tools primarily involve electromyography (EMG) and isokinetic dynamometry of the knee joint. Subjective fatigue was evaluated with patient self-reports. Factors that influence fatigue in these patients include demographic characteristics, sociological factors, sleep disturbances, comorbidities, and disease-specific factors. Interventions included aerobic exercise, rehabilitation therapist-guided home rehabilitation programs, and cognitive behavioral therapy.  Conclusion: This scoping review underscores the need for future large-scale longitudinal studies on fatigue in acromegaly. Priority areas include identifying predictive markers, understanding pathophysiological mechanisms, evaluating targeted interventions, and developing standardized assessment tools to improve early recognition and management.  **Keywords**:acromegaly, fatigue, scoping review, EBN, evidence-based nursing |
| Structured summary |  |  |
| Introduction |  |  |
| Rationale | 3 | With the recent advancements in medical technology and the growing focus on rare diseases, comprehensive clinical biochemical assessment standards have been established for this patient population. However, attention to patients' subjective experiences, particularly self-reported fatigue, remains insufficient among clinical practitioners. Most existing assessment tools primarily focus on generic quality of life measures. Increasing evidence suggests that biochemical control alone does not alleviate the clinical symptoms in these patients. Therefore, investigating fatigue in individuals with acromegaly is essential. |
| Objectives | 4 | population or participants:acromegaly  Concepts:fatigue  The primary objectives of this research are to explore the following issues: 1) the current state of fatigue in patients with acromegaly; 2) the factors contributing to fatigue and their associated adverse impacts; 3) the tools used for assessing fatigue; and 4) the interventions currently employed worldwide to manage fatigue in these patients. |
| Methods |  |  |
| Protocoland registration | 5 | This scoping review was not registered. |
| Eligibility Criteria | 6 | The inclusion criteria were as follows: (1) Studies were included  if participants with acromegaly accounted for ≥50% of the study  sample; (2) studies focused on fatigue-related outcomes; (3) studies  published in both Chinese or English; (4) original research articles.  The exclusion criteria were as follows: (1) studies for which full-text  articles could not be obtained; (2) duplicate publications; and (3)  newspaper articles, comments, and conference abstracts that were  not included. |
| Information sources* | 7 | We searched five international databases (PubMed, Embase,  CINAHL, Web of Science, and the Cochrane Library) and three  Chinese databases (CNKI, WanFang, and Sinmed) from inception  to June 21, 2025. Furthermore, the reference lists of the included  studies were systematically reviewed to identify any publications  that may have been overlooked; these publications were then  included in the analysis. No additional studies were identified in  this research. |
| Search | 8 | e.g.Pubmed:  (((Acromegaly[MeSH Major Topic]) OR ("Growth Hormone-Secreting Pituitary Adenoma"[MeSH Major Topic])) OR (((Acromegaly[Title/Abstract]) OR ("Growth Hormone-Secreting Pituitary Adenoma"[Title/Abstract])) OR ("GH Pituitary Adenoma"[Title/Abstract]))) AND (((((Fatigue[MeSH Major Topic]) OR ("Fatigue Syndrome, Chronic"[MeSH Major Topic])) OR ("Mental Fatigue"[MeSH Major Topic])) OR ("Emotional Exhaustion"[MeSH Major Topic])) OR ((((fatigue[Title/Abstract]) OR ("Fatigue Syndrome, Chronic"[Title/Abstract])) OR ("Mental Fatigue"[Title/Abstract])) OR ("Emotional Exhaustion"[Title/Abstract]))) |
| Selection of sources of evidencet | 9 | To increase consistency among reviewers, all reviewers screened the same 10 publications, discussed the results and amended the screening and data extraction manual before beginning screening for this review.Two reviewers independently extracted data from the included studies.We resolved disagreements on study selection and data extraction by consensus and discussion with other reviewers if needed. |
| Data charting process | 10 | Two master's students, trained in evidence-based nursing, performed an initial screening based on the inclusion and exclusion criteria, followed by a full-text review for re-screening. In cases of disagreement, a third researcher was consulted to determine whether a study should be included. |
| Data items | 11 | Author(s), publication year, country of origin, study design, diagnosis, sample size, duration of intervention, assessment measures, research topic, and other relevant characteristics. |
| Critical appraisal of individual sources of evidence | 12 | Not Applicable |
| Summary measures | 13 | Not Applicable |
| Synthesis of results | 14 | We grouped the studies by the types of behavior they analyzed, and summarized the type of settings, populations and study designs for each group, along with the measures used and broad findings. |
| Risk of Bias Across Studies | 15 | Not Applicable |
| Additional Analyses | 16 | Not Applicable |
| Results |  |  |
| Selection of Sources of Evidence | 17 | A total of 584 articles were identified in the literature search. After deduplication, a total of 433 citations were identified from searches of electronic databases and references of review articles. On the basis of the title and abstract, 308 articles were excluded, and 125 full-text articles were retrieved and evaluated for eligibility. Of these, 105 were excluded for the following reasons: 99 studies were excluded for being irrelevant to the research topic, 5 articles could not be accessed in full, and one study was excluded because it was published in a non-Chinese or non-English language. The remaining 20 studies were considered to meet the requirements of this review. |
| Characteristics of Sources of Evidence | 18 | A total of 20 studies focused on fatigue in patients with acromegaly, with the majority conducted in Brazil (n=7) and the Netherlands (n=3). Other contributing countries include the United States (n=2),China (n=1), France (n=2), Iraq (n=1), Turkey (n=1), Greece (n=1),Canada (n=1), India(n=1),representing 10 countries in total.The basic characteristics of these studies are summarized in Table 1, which provides information on the authors, publication year, country, study design, participant population, sample size, intervention strategy, duration, assessment tools, and distinctive features of fatigue. |
| Critical Appraisal Within Sources of Evidence | 19 | Not Applicable |
| Results of Individual Sources of Evidence | 20 | Details are provided in Table 2. |
| Synthesis of Results | 21 | A total of 20 studies focused on fatigue in patients with acromegaly, with the majority conducted in Brazil (n=7) and the Netherlands (n=3). Other contributing countries include the United States (n=2),China (n=1), France (n=2), Iraq (n=1), Turkey (n=1), Greece (n=1),Canada (n=1), India(n=1),representing 10 countries in total. |
| Risk of Bias Across Studies | 22 | Not Applicable |
| Additional Analyses | 23 | Not Applicable |
| Discussion and Funding |  |  |
| Summary of Evidence | 24 | In this scoping review, we identified 20 key studies published between 2004 and 2025, focusing on the risk factors, assessment, and intervention strategies for fatigue in patients with acromegaly. Our findings indicate a high prevalence of fatigue among acromegaly patients, coupled with a notable lack of attention to this issue. Additionally, we observed that most intervention studies are small-scale, single-center investigations, with a distinct absence of large, longitudinal, evidence-based research. |
| Limitations | 25 | Although this study conducted a rigorous process of literature search and data selection, existing studies on fatigue assessment in patients with acromegaly often combine fatigue with quality of life assessment Many studies have indirectly confirmed the occurrence of fatigue through decreased quality of life, and such literature was not included in this study. Furthermore, fatigue in patients with acromegaly may include both physical and psychological components. Although this study involved these two aspects, there may still be gaps in the covered literature. Finally, our literature search was limited to studies published in English and Chinese, which may introduce publication bias. |
| Conclusions | 26 | Healthcare providers should recognize the impact of fatigue on the physical recovery and quality of life of acromegaly patients. In addition, risk factors associated with fatigue, including disease-specific factors, sleep disorders, and comorbidities, should be identified. Therefore, it is recommended that fatigue be incorporated into the routine perioperative assessment of acromegaly patients. Fatigue should be evaluated both preoperatively and postoperatively, with a focus on tracking changes in fatigue levels. This will provide valuable data for future studies on fatigue trajectory in these patients and facilitate timely identification and intervention, ultimately promoting faster recovery. |
| Funding | 27 | This study was supported by the Zhejiang Medical and Health Science and Technology Program under program number 2024KY1031. |
