# Supplementary material for: Fatigue in acromegaly patients: a scoping review
Source: Front Endocrinol (Lausanne). 2025 Jul 14;16:1601661. doi: 10.3389/fendo.2025.1601661 (PMC12301152; doi:10.3389/fendo.2025.1601661)
Supplement: Supplementary file 2 [file DataSheet2.docx]

Appendix 2

e.g.Pubmed:

(((Acromegaly[MeSH Major Topic]) OR ("Growth Hormone-Secreting Pituitary Adenoma"[MeSH Major Topic])) OR (((Acromegaly[Title/Abstract]) OR ("Growth Hormone-Secreting Pituitary Adenoma"[Title/Abstract])) OR ("GH Pituitary Adenoma"[Title/Abstract]))) AND (((((Fatigue[MeSH Major Topic]) OR ("Fatigue Syndrome, Chronic"[MeSH Major Topic])) OR ("Mental Fatigue"[MeSH Major Topic])) OR ("Emotional Exhaustion"[MeSH Major Topic])) OR ((((fatigue[Title/Abstract]) OR ("Fatigue Syndrome, Chronic"[Title/Abstract])) OR ("Mental Fatigue"[Title/Abstract])) OR ("Emotional Exhaustion"[Title/Abstract])))
